# “Are You a TA Practitioner, Then?” – Identity Constructions in Post-Normal Science

**DOI:** 10.1007/s11024-022-09480-x

**Published:** 2022-12-05

**Authors:** Karen Kastenhofer, Anja Bauer

**Affiliations:** 1 Institute of Technology Assessment, Austrian Academy of Sciences, Vienna, Austria; 2 Dpt. of Science Technology and Society Studies, University of Klagenfurt, Klagenfurt, Austria

**Keywords:** Academic Identity, Academic Generations, Transdisciplinarity, Technology Assessment, Scientific Community, Post-Normal Science

## Abstract

Technology assessment (TA) is a paradigmatic case for the manifold and, at times, ambiguous processes of identity formation of researchers in inter- and transdisciplinary settings. TA combines the natural, technical, and social sciences and follows the multiple missions of scientific analysis, public outreach, and policy advice. However, despite this diversity, it also constitutes a genuine community with its own discourses, conferences, and publications. To which extent “being a TA practitioner” also provides for a genuine scholarly identity is still unclear. Building on interviews with technology assessment practitioners at an academic TA institute, we ask what inter/trans/disciplinary identification patterns emerge in this field. Our analysis shows that TA practitioners adopt multiple identities, from “enthusiastic TA practitioner” to “strong interdisciplinarian” or “disciplinarian” – with distinct identity troubles inherent in all these options. We find that generational affiliation plays a vital role in identity formation. It relates to different primary orientations (towards research or advisory practices), inter/disciplinary backgrounds, contracting modes, and lengths of time spent at the TA institute. We conclude firstly, that disciplinary categories figure strongly in transdisciplinary identities; secondly, that the relation of *chronos* and identity warrants more substantial consideration: as time spent at a transdisciplinary institute as or as perceived options for “futuring one’s identity”; thirdly, that our understanding of academic generations could profit from a more sociological conception; and, fourthly, that TA’s multidisciplinary setup and threefold orientation towards science, society, and policy result in multiplying possible identities and thus making it difficult to form a stable community.

## Introduction: A Focus on Academic Identities

Science and technology studies, history of science, and higher education research have long acknowledged that academic scholarship and its disciplines represent temporary entities resulting from ongoing processes of co-construction, recalibration, and stabilization (e.g., Gieryn 1983, on the strategic demarcation of science). History of science scholars have described how the modern disciplinary structure of science is a relatively new phenomenon and might already be changing its character once more (e.g., Stichweh 1992). Higher education research has outlined the socializing and enculturating effects of disciplinary curricula, in accord with the twofold meaning of the term “discipline” (Huber and Vogel 1984; Beaufays 2003; Arnold and Fischer 2004). Science and technology studies scholars have highlighted how different disciplines and subdisciplines come with distinct epistemic cultures (Knorr-Cetina 1999) linked to specific epistemic communities (Bulpin and Molyneux-Hodgson 2013) and evidence-based regulatory regimes (Haas 1992).

Throughout all these analyses and insights, one central aspect has mostly escaped scrutiny: How do scientists (including not only natural scientists, but also engineers, social scientists, and humanities scholars) come to identify as such and/or as members of a discipline or an epistemic community? This question not only touches on the more practical aspects of initiation and subjectivation processes, but also relates to our very conception of science and its disciplines; especially when patterns and regimes of identification are shifting in societies at large and identities become more “liquid” (Baumann 2000), evasive and hybrid, while also gaining momentum as strategic resources of identity work and identity politics (Wetherell 2010). What kind of science without (self-declared, perceived, felt) scientists and scientific institutions? What sort of disciplinary community without (self-declared, perceived, felt) relating disciplinarians and disciplinary institutions? A renewed focus on shifting modes and patterns of identification in contemporary science seems warranted. These modes and patterns become particularly relevant in the context of interdisciplines and transdisciplines institutionalized during the end of the 20th century or emerging at the beginning of the 21st century (cf. Kastenhofer and Molyneux-Hodgson 2021), as such fields integrate various disciplines to provide for novel technoscientific approaches (interdisciplines) or to integrate societal concerns and actors (transdisciplines).^1^

In this article, we focus on inter/trans/disciplinary patterns of identification within a transdisciplinary field that goes back to the 1970s, namely technology assessment (TA). TA exhibits characteristics of “post-normal science” (Funtowicz and Ravetz 1993) that provides expertise on societal issues with high uncertainty and significant decision stakes. Based on interdisciplinary (and partly participatory) research, TA responds to the quest for evidence-based and democratic decision-making in the governance of emerging technologies. TA is thus oriented towards academic research as well as public outreach and policy advice. Technology assessment also differs from classical disciplines as it is not taught in respective university curricula but acquired and grown into throughout post-graduate, hands-on experiences. Nevertheless, TA does not represent a singular case; rather, it is an example of a new type of mission-oriented scholarship that keeps gaining importance in the societal quest to increase sustainability and responsibility (cf. OECD 2021).

Our analysis of identification patterns in TA adds to previous studies of the relation of TA to its clients (Kastenhofer et al. 2019) and the repertoire of advisory roles of TA (Bauer and Kastenhofer 2019). It builds on significant insights from the literature (see next section) for explanatory arguments and further conceptual refinement. Section 3 introduces our empirical case, the Austrian Academy of Sciences’ Institute of Technology Assessment (ITA) and outlines our methodological approach. Section 4 presents the main results of our research: we reconstruct how identity options and moments of identification vary along three generations of TA practitioners at ITA and along the practitioners’ various disciplinary backgrounds. In the conclusion, we discuss what these findings add to our understanding of identity constellations in contemporary academia more generally and in advisory science more specifically.

## Inter- and Transdisciplinary Identities: The Role of Disciplinary Contexts, Institutional Forms, and Temporalities

Within science and technology studies and higher education research, the scholarly literature is ripe with hints of identity challenges in contemporary academia but does not feature a concerted debate about academic identities. There is talk of “provisional selves” (Ibarra 1999), “troubled identities” (Cuevas-Garcia 2021), and “identity schisms” (Winter 2009). There is also debate about “choreographing identities” (Schikowitz 2021) as a strategic, career-oriented response (or “careering”, cf. Clarke and Knights 2015). Addressing contemporary higher education and research regimes more broadly, some scholars proclaim an era of "post-disciplinarity" (Frickel 2004), while others assert that disciplines "have retained much of their normative power" (Henkel 2005:155) and still constitute the dominant mode of structuring science. More recently, Cuevas-Garcia (2021) confirms that identity trouble resulting from interdisciplinarization is associated with more heterogeneity, but not with an abandonment of disciplinary narratives. Accordingly, Marcovich and Shinn (2011) suggest speaking of a “new disciplinarity”, characterized by a “deep-seated transformation inside scientific disciplines, where disciplines nevertheless retain foundational features of their historical identity and functions”. (ibid: 584). Overall, disciplinary categories are depicted as challenged but not obsolete points of reference for subjectivation processes, community building, career development, and identity work.

This discussion of historical shifts of academic inter/disciplinarity should be accompanied by consideration of the recent proliferation of institutional ambitions and organizational modes in higher education and research: not only the inter/disciplinary character of organizational units in academia has diversified but also their mix of disciplines, their temporal scope (permanent, open-ended, or temporary), their time horizon (one year, six years, or longer), their institutional form (project, institute, community, or field), their transdisciplinary setup (purely academic or hybrid), and their ambitions (education, networking and/or research, maximum scientific output, maximum marketable innovation, or transformative societal intervention). Technology assessment shares some characteristics with, but also differs from, some other interdisciplinary constructs: emerging technosciences (such as systems biology or bioinformatics as researched by Calvert 2010; Kastenhofer 2013; Bartlett et al. 2016); stabilized interdisciplines (such as environmental science or sustainability science (as researched by Müller and Kaltenbrunner 2019; Parker and Hackett 2012; Felt et al. 2013, 2016; Schikowitz 2021); temporary collaborations between scientists, engineers, and liberal arts scholars fostering more responsible research (e.g. Balmer et al. 2015) or inducing more profound innovation (e.g. Bock von Wulfingen 2021); and transdisciplinary boundary organizations (e.g. Parker and Crona 2012).

Among other themes, this literature addresses the major issue of junior researchers growing into an inter/trans/disciplinary identity. While earlier higher education research has provided us with a refined understanding of socialization, enculturation, and subjectivation during undergraduate studies in disciplinary curricula, this recent work focuses on inter- and transdisciplinary post-graduate settings. Felt et al. (2013, 2016) highlight the difficulties that PhD students face when trying to construct consistent and sustainable identities against the horizon of a six-year sustainability science program that included a doctoral school (Felt et al. 2013). Core challenges related to orientation, attaching, and positioning, all of which were closely interwoven with questions of identity (Felt et al. 2016; Schikowitz 2021). Bock von Wülfingen (2021) presents the case of identity transition within a multidisciplinary Cluster of Excellence in Germany, that was also funded for six years. For this case, the author discerns different time horizons of identity formation: on a *short-term* basis, alternative categories such as academic status, family background, and professional self-esteem gained prominence in interactions as well self-perceptions, while, after a certain amount of time, the “the lack of a disciplinary community was (…) compensated by belonging to the broader interdisciplinary Cluster community”. She thus notes that previously acquired academic and social status can have a stabilizing effect during inter/trans/disciplinary identity transitions.

For mid-career inter- and transdisciplinarians, the more important challenge becomes sustaining a career and identity rather than problems with gaining an identity and growing into a community. Müller and Kaltenbrunner (2019) focus on a permanent environmental science research centre founded in the mid-1990s at a Swedish university (and thus comparable to the ITA). In their case study, science policy discourses and funding programs increasingly support interdisciplinary careers but these careers keep being severely challenged as “the overriding power of formal career incentives at universities (…) continues to emphasize the ideals of the individual high-performing academic who publishes in disciplinary journals and attracts the most selective funds” (ibid: 479). The authors devised three roles exhibited in this ambivalent situation: the “reliable researcher”, the “politically correct researcher”, and the “residual slacker”, all of which “enact and reinforce a value hierarchy” along which “interdisciplinary work (…) becomes possible, but (…) always represents the less prestigious counterpart to disciplinary research” (ibid: 495-6).

Parker and Crona (2012) present a comparable case study on the Arizona State University’s Decision Center for a Desert City. This unit was established as a boundary organization in the alternative academic milieu of The New American University, ”with the goal of contributing to basic research (…), while also working closely with resource managers and policymakers to enhance long term decision-making”. The authors identify four tensions that need to be addressed at the boundary between academia, public outreach, and policy advice – interdisciplinarity vs disciplinarity, long-term vs real-time, basic vs applied, and autonomy vs consultancy. Thus, the major challenge is not to answer to uniform ideals of academic excellence, but to accomplish temporal stability, which is “achieved only occasionally in relation to changing organizational environments, resource availabilities, and stakeholder demands" (ibid: 284). In their analysis of the emergent Resilience Alliance, “a tightly networked coherent group” of sustainability scientists collaborating in an academic setting, Parker and Hackett (2012) report on how distinct formats (like “island time”, "structured around informal, highly personal relationships, engendered in part by the fact that some [Resilience Alliance] members have known each other for three decades") foster community building, whereas the diversity of participants and the movement's sheer size "[had] the potential to erode the affective culture that generated initial successes". Scholars such as Balmer et al. (2015), Bartlett et al. (2016), or Jacob and Jabrane (2018) address stabilization via division of work or functional differentiation among interdisciplinary scientists with different disciplinary backgrounds.

A final important strand of case studies focusses on the parallel emergence of new technoscientific fields and respective scholarly identities. Pertinent cases again exhibit distinct temporal characteristics. As *fields*, the new technosciences are thought to persist without expiration date; as *emerging* fields, they are considered as still developing and meant to undergo typical phases, like "birth" or emergence, establishment, maturation, sometimes also "death". This developmental view has been most prominently introduced by Mullins’ (1972) paper on the development of molecular biology. More recently, Calvert (2010) rekindled the discussion of identity constellations in emerging technosciences with her study on systems biology. With this emerging field, it became particularly apparent that “identity has a trajectory – it is connected to the past and the future” (Wenger 2000), via past education, training and career steps, future identity aspirations, or assumed future job opportunities for junior scientists. Bartlett and colleagues (2016) further refine the distinction between junior and senior scientists by referring to distinct generations within bioinformatics (also described in Kastenhofer 2013). In their reconstruction of three generations, they borrow from Ben-David and Collin’s (1966) distinction of forerunners, founders, and followers and their analysis connects with a kind of disciplinary biography (marriage, birth, and childhood) prevalent in bioinformatics narratives. Bartlett and colleagues conclude “that the cultural differences at play in interdisciplinary work are not only those of different disciplinary cultures, but also ‘generational’ differences” (ibid: 192).

Our analysis and discussion of identities in technology assessment is informed by this rich literature: TA can be understood as a movement, as well as a constantly restabilizing interdiscipline. The Institute of Technology Assessment comes with its own pasts and futures. Its scientific staff was hired during different developmental phases of the institute. The shifting constellations likely had an impact on identity formation, as did the diverse inter/disciplinary identities that the individual scholars had acquired before joining the institute. Moreover, the difficulties for different career stages, as outlined in the literature, can be expected to pertain to our case: the “identity trouble” that comes with moving to a transdiscipline, the challenges that come with growing into a transdisciplinary identity, and the efforts of sustaining it throughout one’s career. Particularly the insights on “identity as a trajectory” and generational belonging also sensitized our empirically grounded analysis. In discussing what types of identities had been formed, we additionally compared our empirical case (TA and the Institute of Technology Assessment) with various other modes of institutionalization and organization prevalent in the previously mentioned case studies, further refining our explanatory arguments. Against the background of this empirically rich literature, we could thus ask what identities had been adopted in TA and how processes of identity formation and adaptation relate to distinct individual and institutional characteristics and requirements.

## Material and Methods: An Empirically Grounded Case Study

Our analysis focuses on TA as a post-normal scientific practice. TA has been defined as “an analytic and democratic practice that aims to contribute to the timely formation of public and political opinion on societal aspects of science and technology” (van Est & Brom 2012: 306) or as “a field of research and practice of scientific policy advice on socially sensitive technology issues” (Nentwich and Fuchs 2021: 4). TA combines natural, technical, and social sciences and follows the missions of scientific analysis, public outreach, and policy advice. On these grounds, it is a paradigmatic case for the manifold and, at times, ambiguous processes of identity formation and transformation of researchers in inter- and transdisciplinary settings. TA is not an academic discipline open for enrolment at universities. Scholars are not socialized and encultured in this field during their primary university education. The first organization to formally provide TA was the Office of Technology Assessment (OTA), established in 1974 by the U.S. Congress. Although OTA was closed in 1995, it continued to serve, from the 1980s, as a model for Europe’s flourishing and diverse TA landscape, affiliated with parliaments or academic institutions at national and transnational levels. Of late, few TA institutes have been closed (in Denmark temporarily in 2011, in Italy and in Flanders/Belgium in 2013), while TA has further proliferated to Asia and Latin America. Overall, the field of TA is therefore described as both “dynamic” and “fragile” (Böschen et al. 2021: 14).

Our discussion of identity constructions in technology assessment builds on a study of the Institute of Technology Assessment (ITA) at the Austrian Academy of Sciences (OeAW). ITA is the primary TA institution in Austria, "deal[ing] with the impacts of new technologies on society, the environment and the economy. It carries out scientific technology assessments on a variety of topics. The results of this work support policymakers, administration and the public with regard to issues of technology policy.”^2^ ITA was officially established as an institute of the OeAW in 1994, following a 10-year period as a temporary working group or research unit tasked with TA-related issues. It is thus primarily a scientific organization, balancing research and advisory activities. Over the past 30 years, the institute has undergone several developments in its approaches, advisory activities, contractors/clients^3^, and addressees and staff (for more details, see Nentwich and Fuchs 2021, as well as the next section of this paper).

Of ITA’s twenty researchers at the time of our study, three had graduated in sociology (two with additional post-doctoral qualifications), five combined a social science (economics, political science, sociology, communication science) with STS, one combined a legal doctorate with a post-doctoral qualification (“Habilitation”) in STS, four combined a doctorate in biology with further qualifications in STS and/or human ecology, one a doctorate in chemical engineering with a post-doctoral qualification in TA and sustainability science; three came with a double qualification (biology plus archaeology, history, or philosophy), three stem from economics, management, or informatics. In sum, thirteen held a doctorate, and four had additionally completed a post-doctoral qualification. ITA’s scholarly staff is not split into formal sub-units; formal hierarchies only pertain to a differentiation between scholarly staff and directorate. There are, however, changing thematic areas and leadership positions in distinct projects.

Our analysis is based on a series of interviews, including all twenty researchers working at ITA at that time (March and April 2017). Interviews lasted between ninety minutes and three hours. They were prepared, conducted, and analysed in tandem by the two of us, including two interviews with each other, as we were both also members of ITA.^4^ With this general setup, our study followed a twofold "research from within" approach (Sikes and Potts 2008; Trowler 2011; for a more detailed discussion of applying this approach to this specific project, see Bauer and Kastenhofer 2019): When researching inter/trans/disciplinary identity *in* academia *as* academics, we observed and made sense from distinct positions and positionings within this very same (academic) realm. Since we also analysed our own research organization, this demanded additional careful preparation, systematically taking into account our own biases, power relations and strategic interests by implementing extensive intra-organizational feedback cycles. For the theme at hand, the diversity within the team of interviewers as to disciplinary backgrounds (biology / political science) and time spent at ITA (10 years / 2 years) served as an important point of reference. With a shared identification with interdisciplinarians of the second generation, we invited “enthusiasts” and “disciplinarians” from the first and “flexible practitioners” and “early interdisciplinarians” from the third generation to comment on our findings (for all these types, see the next section).

Another important feature of this project is its empirically grounded approach. During a series of interviews focusing on advisory practices in TA, we could not ignore the interviewees’ continual references to issues of identity, career, and belonging. We thus decided to extend our research focus and to methodically introduced the explicit question "Are you a TA practitioner, then?" in the last section of the interviews. The transcribed interviews were analysed following Grounded Theory (Corbin and Strauss 2008) with the help of MaxQDA software. We built on the interviewees’ unprompted references to identity during the first sections of the interviews as well as on their explicit reactions to our direct question in the last section. The various analytical steps are outlined below. All interview quotes have been translated by the authors; any identifiers have been omitted to guarantee the anonymity of all interviewees.

## Patterns of Identification Within Three Generations of TA Practitioners at ITA

Our question about the interviewees’ identity evoked remarkably diverse reactions. Some were surprised about the need to even ask the question; others relativized or rejected the opportunity to identify directly as a TA practitioner and provided alternative identifications. While the diversity of reactions seemed interesting in itself, we also searched for distinct factors that allowed for a deeper understanding of patterns and logics of identification in a post-normal science context. After assembling additional material on our interviewees’ careers, we detected two influential dimensions: on the one hand, the interviewees’ disciplinary backgrounds; on the other hand, the year they had joined ITA. Also, we detected a particular relationship between these two dimensions. In the next step, to help better understand the emergence of distinct generations, we researched the history of the institute and changes in its direct environments.^5^

In the following, we present distinct types of inter/trans/disciplinary identification that we found in the material. Each generation is introduced along with its most characteristic (and, to some extent, dominating) type of identification, complemented by additional types and followed by historical explanations for the emergence of these types.

### The Founding Generation: “TA enthusiasts”, “Disciplinarians”, and “Feeling Like a Stranger”

*[Are you a TA practitioner, then?] Yes. (…) Because I have been practicing TA for decades, because it interests me, because technology is very influential. Originally, I was fascinated with technology, and interest in the political, the societal aspects only followed later onwards. If one can contribute to elucidating societal conflicts, that is highly motivating. My identity did not change that much. With time, one loses one’s bonds to one's [educational] discipline; but my interests never lay that much with [this discipline], anyways. With my interest in political issues, TA is almost a better fit than [other institutional places closer to his/her university education].* 6

The above quote represents the most straightforward type of identification with TA in our material. It fits with interview statements like “certainly I am a TA practitioner!” and public self-representations as “enthusiast in the field of technology assessment”, “passionate technology assessor”, or “associate professor in the field of technology assessment"^7^. This strong identification with TA is coupled with only residual ambiguity (like holding a PhD or postdoctoral qualification in some other field). We subsume this sub-group of interviewees^8^ under the label “TA enthusiasts”. They had gathered expertise in bridging basic research and applied questions, technoscientific and societal issues, either via a multidisciplinary training or job experiences outside of academia prior to joining ITA. They were among the first to officially perform TA in Austria, joining ITA during its pre-foundational and foundational phases (from 1988 to around 2000). The apparent absence of major identity struggles can partly be explained by their permanent and central institutional positions when they were interviewed (or, in other words, their identity thus being “future-proof”), but also by their prior inter/trans/disciplinary experiences before engaging with TA and the historical situation when joining ITA.^9^

During ITA’s foundational years, ITA practitioners were recruited based on their specific disciplinary expertise such as sociology, economics, law, information and communication technology, engineering, biology, and health science to ensure interdisciplinarity. Yet, in their TA activities, they generally focused on analysing specific technologies (such as information and communication technology, energy and environmental technology, health technology, or biotechnology). These technologies were addressed in a comprehensive manner with the aim to cover all aspects potentially relevant to society or from a distinct perspective (such as privacy, equity and justice, health risks, or environmental impact) or position (such as consumer rights). First-generation scholars were thus not only defined by their “home discipline”, but also recognized internally and publicly as experts for one distinct technological field. This public recognition and identification as “experts for [X]-technology" was fostered by a relatively strong orientation towards advisory activities for Austrian ministries and the national council (as compared to basic research funded by Austrian or European science funding agencies). The small size of the institute at the time and the joint effort to establish a new institution within Austrian academia and the technology governance landscape resulted in strongly intertwined feelings of collegiality, camaraderie, and friendship. Rather than passively adopting a given TA identity – an identity not yet established in Europe, with only the U.S. Office of Technology Assessment (1974–1995) as the main point of reference – the ITA staff independently shaped what TA was meant to be in the Austrian context. They actively did so with each new project, defining TA indirectly as the sum of all activities performed at ITA. All the more important it was to pursue the “right” projects and conducting them in a way that fit with all ITA members’ visions of TA. Repeated arguments about how to practice TA at ITA testified to this situation. Despite all these struggles, the overall institutionalization of TA and the ITA in Austria formed a first generation of TA practitioners that strongly identified with their institute and their newly established field and practice.

However, not all first-generation practitioners shared the same level of identification. Some portrayed themselves as more distant to TA and preferred identifying with their original discipline. We subsume this group under the label “disciplinarians”. They identify as “a trained [disciplinarian] who works in technology assessment” or as “someone who comes more strongly from the scientific side”, “attaching primary importance to a scientifically sound, methodically robust basis” in their practice. Within this group, two interviewees point towards troubling feelings of alienation and of sitting uncomfortably between the two chairs of TA and their original discipline. This latter type of (non-)identification we subsume under the label of “feeling like a stranger” characteristic of interviews with “disciplinarians” of the first generation with a background in technoscience.

Other than the “TA enthusiasts”, “disciplinarians” identified primarily as scientists, explicitly distancing themselves from more “activist” stances^10^ and voiced an ongoing concern about how to preserve objectivity in TA practice and as a public persona. Coping strategies of this disciplinarily mixed group included taking up a more passive (making protocols, summarizing the state of affairs), analytical (providing statistical evidence), or facilitating (moderating workshops) role. The sociologists within this group refrained from engaging in public discourse with pointed commentaries because “that [was] not who [they were]”. But while their explicated ideals of evidence-based, objective policy advice resembled those of the natural scientists and engineers, they did not recount many problems in finding their role within policy advisory settings that required dealing with distinct issues and stakeholder positions. This may have been because they had already been aware of the complexities and ambivalences of advisory practice and had the relevant expertise to manoeuvre in such contexts.^11^

The scientists and engineers frequently mentioned the sudden shift from being an established member of a disciplinary community to being a transdisciplinarian. They recounted loyalty conflicts, role uncertainty, position ambivalence, multiplication of identities, and epistemic insecurity. They still grieved about the loss of their former identity and identity options, or, in other words, that, for them, "science [as a career and identity option] had died”. These struggles were exacerbated by the palpable politicization, even within academia, and the heated technology controversies and polarizations in Austria during the institute’s foundational phases. One former technoscientist^12^ reported on feeling like a stranger (before "finding their own niche") and also that their individual political positioning (or, in their perception, “political abstinence”) disappointed expectations of both some new colleagues at ITA (being seen as too uncritical of emerging technologies) and public players from their former technoscientific community (being accused of denigrating their discipline^13^). Sub/disciplinary identity and belonging were also felt in internal collaborations, just as bio-ecologists and molecular biologists clashed in their takes on agro-biotechnology risk assessment or technical physicists and medical experts would clash in their takes on health risks posed by electromagnetic fields (for both well-researched clashes of epistemic cultures, cf. Böschen et al. 2010). Another former technoscientist was less ambiguous, but accounted for holding multiple identities: “Yes, I am a TA practitioner. But in my private life, I am still regarded a [technoscientist] and think like a [technoscientist].” In a later phase, they tried to make up for their perceived lack of sociological expertise:

We had contacts with a group at the university of economics that dealt with ecological economics; that led me [as a technoscientist] to philosophy. I also read Luhmann for the first time in my life, just to see what sociologists added to the picture. And I tried to understand, with help from my institute, and with plenty of discussion. Not as to be able to apply it, but as to better understand.

The natural scientists and engineers thus shared a certain feeling of estrangement and of having to become a different kind of expert. The various coping strategies came with their own insecurities as they still needed to acquire the necessary (statistical) methods, (facilitator) skills, or (theoretical) insights. They also reported on changing self-perceptions, that while “initially, [they had been] convinced to be very objective”, they became more cautious after having experienced, with significant irritation, the simple impossibility of keeping an objective stance.

Overall, we could thus reconstruct three types of (non-)identification with TA: (1) outright identification with an enthusiastic streak (“TA enthusiasts”); (2) the disciplinarian disposition, concentrating on scientifically sound empirical work and refraining from a more activist stance (“disciplinarians”); and (3) a sub-type of the “disciplinarian”, feeling estranged both from the former technoscientific community as well as the newly forming group, (“feeling like a stranger”). For this generation of TA practitioners, the first type featured most prominently as a common point of reference.

### The Interdisciplinary Generation: “Strong Interdisciplinarians”, “Embedded TA Practitioners”, and “Disciplinarians”

*Over the years, I have already been at several institutions; my socialization was in [an interdiscipline* 14 *]; [this interdiscipline] somehow is my home; I am a [social/natural] scientist. Technology assessment has always been the focus. My disciplinary community certainly is [the interdiscipline]. On the other hand, also [a thematic] community. These are my main communities.*

As this quote illustrates, most of the seven members of a second generation of ITA members had already acquired a professional identity before joining ITA; one they did not perceive as that different or as less valid than an outright TA identity. They could coherently construct their identity path and career, comparable to the first-generation “TA enthusiasts”, but they explicitly identified as “belong[ing] to the interdisciplinary generation”. Also, they were much more self-assured about their academic status than the first-generation “disciplinarians”. If the former scientists and engineers had gone through “painful identity transformations” to being an “interdisciplinarian” such as a human ecologist, sustainability science or science and technology studies scholar, the second-generation practitioners had completed these transitions long before joining ITA and engaging with TA. Those with a social sciences background would depict these same interdisciplinary fields as specializations or subcommunities of their original fields and thus did not need to undergo major transformations of their identity. However, many felt challenged by their disciplinary or interdisciplinary environments, which did not always recognize TA as an academic field, as well as by fellow ITA members who occasionally questioned their degree of identification with TA. Moreover, they could not count on either their original discipline or TA as being known to the public and therefore they chose alternative modes of identification in everyday conversations: “Most times I present myself as a technology analyst and social scientist and I then add that this is TA, at ITA. Because it is easier to grasp that way.”

The strong presence of interdisciplinarians in the second generation of TA practitioners can be traced back to historical developments in ITA’s environments. Firstly, academia and academic research funding had undergone a period of inter- and transdisciplinarization in the wake of environmental concerns and technology controversies. Interdisciplinary institutes and departments, founded like ITA in Austria in the 1980s, had come to represent new fields such as human ecology, ecological economics or science and technology studies. The higher education system had evolved to provide for interdisciplinary curricula bridging the divide between technosciences and social sciences and humanities. These courses were mostly institutionalized as post-graduate master's programs and accepted students from diverse disciplinary backgrounds. At the international (European Commission) and national (Austrian Research Promotion Agency) level, programs (focusing on ethical, legal, and social aspects of technology) began funding research to prevent future controversies, polarizations, and stalemates related to technology. Secondly, the Austrian Academy of Sciences underwent in 2007 a fundamental reform resulting in international scientific advisory boards, a more managerial leadership style, and a stronger focus on competitive research.^15^ At almost the same time, a new employment law at Austrian academic institutions (*Kettenvertragsregelung*)^16^ led to a de-facto limit of six years for consecutive contracts of TA practitioners at ITA, curbing their future career prospects at ITA and opportunities to pursue TA-related identities. With insecure career prospects at the only Austrian TA institute, the second generation remained strongly oriented towards interdisciplines that then offered more career options.^17^ Also, identity work at the institutional level changed as the new criteria of excellence did not seem to adequately reward the institute’s quest for interdisciplinary integration, accessibility, inclusion, and societal impact. Safeguarding a distinct TA identity while securing the standing of the institute within the Austrian Academy of Sciences became central motivations for a dual strategy: on the one hand, conforming to the newly implemented evaluation regime led to an emphasis on research rather than advice, a focus on basic and program research rather than contract research and a reconsideration of the institute’s publication strategy. On the other hand, the institute fought for alternative evaluation criteria, pointing towards its inter- and transdisciplinary character. Thirdly, this phase coincides with a drastic expansion of ITA personnel. The institute’s personnel had been reduced in 2006 when a group of ITA members left the institute to establish the Austrian Institute of Health Technology Assessment. This left existing positions at the institute vacant while new project contracts provided for further positions. From 12 scientists in 2006, the institute expanded to 22 scientists in 2008 (cf. Nentwich and Fuchs 2021).

All three factors help to explain the emergence of a new generation of TA practitioners, in which the distinct identity pattern of “strong interdisciplinarians” played a central role: junior to senior post-docs with disciplinary backgrounds, additional post-graduate interdisciplinary training, experience, and competitive track records in science and technology studies, human ecology, or risk research. The expertise of individual practitioners became even more specific: analysing the relevance of discursive framing on the governance of biotechnologies, providing politically relevant recommendations on privacy issues with information and communication technologies, or organizing participatory workshops on citizens’ visions of a sustainable future. They also started to publish on more abstract issues pertaining to the distinct character of TA, like inter- and transdisciplinarity, advisory practices, or participatory methodology.

But even the identity of the “strong interdisciplinarians” was not untroubled. The opportunity to continue relating to their former interdisciplinary communities after having joined ITA was thought to trigger distrust about their sense of belonging and their enthusiasm for TA with established ITA members. The distinction of a “TA practitioner in a strict sense” or “a narrow sense” came up:

[Are you a TA practitioner?] Is this a “yes” or “no” question? Well, (…), yes, a TA scholar with a broad political conception. I doubt that many at the institute would label me as a TA practitioner in a strict sense – that’s why I hesitated. As to a socio-political mission, comparing my original motivation at the beginning of my scientific career and where I am now, there is some stringency, and in that sense, I do see myself as a TA practitioner. And in the narrow sense, along which I would have to explicate where the TA is to be found in a certain project, I do not see myself as a TA practitioner.

The new influx of interdisciplinarians socialized in very similar fields also triggered questions whether this or that project or practice “still was TA” – and not science and technology studies or human ecology. This kind of boundary work aimed at safeguarding the specific identity and raison d’être of TA in relation to the prevalent interdisciplines. The methodological core of TA was put up for discussion, as was the TA-specific balance between epistemic ambitions and societal missions. In related debates, the different inter/disciplinary backgrounds were again felt vividly. Trained social scientists would emphasize the central role of sociological methods and expertise in TA, while others emphasized the centrality of TA-specific approaches and skills or of perspectives also prevalent in human ecology and ecological economics.

Although the “strong interdisciplinarians” type figured most prominently for this generation of interviewees, other TA practitioners were more strongly affiliated with a distinct line of advisory projects and related networks, approaches, themes, and issues. We subsume this pattern of identification as “embedded TA practitioner”. In such cases, disciplinary or interdisciplinary affiliations seemed less relevant. The second generation also features the “disciplinarian” type comparable to the first generation, but in a more self-assured form. All interviews with members of this generation mentioned that their full commitment to TA was inhibited by temporary employment contracts and unclear career prospects. At the time of the interviews, only two held a permanent position and the academy provided no reliable career scheme.

### The Newly Emerging Generation: “Flexible TA Practitioners” and “Early Interdisciplinarians”

*[Are you a TA practictioner?] With [project X], I did not have this impression, with [project Y] actually neither. With the ongoing [project Z] I would have this feeling, but this is unfortunately only a very short project. But with [project Z], I thought I could identify with TA more easily if this were a larger project.*

Among the youngest generation of TA practitioners, identities seemed most vague and uncertain. These seven scholars’ identities centred around very flexible adjustment to diverse TA project contexts (“flexible TA practitioners”) and still ongoing interdisciplinary specializations (“early interdisciplinarians”). While perceiving ITA as a highly attractive institute that provided for "interesting", "challenging", "independent, yet well-supervised" work in a lively environment with strong social commitment, this generation harboured anxieties that they “lag[ged] behind with [their] identity” or might never gain a clear expert status while hopping from one project to the next. Being an expert in TA was seen as linked to a certain topic or theme – internally as well as externally – and it was something that seemed reserved to senior staff members who identified as “work[ing] on privacy issues”, “do[ing] a few things on big data [and thus gaining] some recognition in this context”, or as thematically “it actually always [having been] the biotech area [for them]” and could also reject projects on these grounds.^18^ The senior practitioners’ expert status came with crucial networks outside the institute that solidified their professional identity by recognizing them as reliable, trustworthy experts. The younger TA scholars naturally lacked these more specialized sources of identity and could only hope to build them eventually. When comparing themselves to their established peers, they did not strongly feel that they had certainly acquired an exceptionally broad range of TA skills and expertise in a relatively short amount of time; this strength was noticed, however, during research stays at other institutions, boosting their self-esteem as well as their identification as TA experts.

That members of the youngest generation of TA practitioners were overall least secure about their inter/trans/disciplinary identity clearly relates to the relatively shorter time they had spent at the institute and their yet unclear career prospects. Again, further contextual factors also became detrimental in the identity configurations of this third generation. Firstly, the Austrian higher education system had further evolved, offering a broad array of pre-graduate interdisciplinary specializations (“modules”), thus blurring disciplinary boundaries even further and providing for a generation of “early interdisciplinarians”. Secondly, interdisciplinary institutes established in the 1980s in Austria and challenged by the 2002 university reform were additionally threatened when the global financial crisis resulted in severe cuts in public budgets. Some eminent European parliamentary technology assessment institutes were severely challenged (e.g., in Denmark in 2011) or closed (e.g., in Belgium in 2013), leaving the overall impression that interdisciplinary institutions and TA institutes “come and go” (or “rise and fall”, Nentwich & Fuchs 2021:39). More specifically, the third generation of TA practitioners had started employment during or right after another struggle to safeguard the institute’s continued existence. A senior member of the institute noted accordingly that "this was a time when the institute (…) again (…) hang in the balance. Because they actually wanted to close it down."

The situation improved only when the Austrian Ministry of Science recommended that the academy as a whole become “more involved in the field of political and societal advice”^19^ and specifically mentioned ITA’s potential role in this reorientation within the performance agreements 2012–2014. For the institute, this official recognition of its dual focus confirmed its own self-image and safeguarded its future (cf. Nentwich and Fuchs 2021). The renewed self-esteem as a policy advisory institute intensified ITA’s positioning not only as an academic interdiscipline, but also as an advisory unit, and, overall, as a field of practice with unique characteristics, distinct from other academic fields. It also fuelled ongoing negotiations with the Austrian Parliament to contract advisory activities with ITA on a regular basis and was reinforced in 2016 with their positive settlement. ITA became a full member of the European Parliamentary Technology Assessment network (EPTA). Its advisory practice gained more relevance, both internally and externally, that nurtured an identity of “flexible TA practitioners”.

With the third generation of TA practitioners, emphasis was thus placed on advisory projects. TA practitioners no longer focused on one thematic working area alone, but also specialized in generic advisory expertise, such as advisory techniques or participatory methods. In this phase, a new generation of practitioners started their career in TA at a relatively early career stage, right after finishing their master’s degree (or *Magister*). While former generations of TA scholars had mostly moved to ITA with a doctorate, this younger cohort could not build on an already established and formally certified (disciplinary or interdisciplinary) identity. They described their start at ITA as an inspiring as well as confusing period of “project hopping”. Completely assuming a TA identity based on a temporary contract continued to pose a considerable risk. Moreover, they learned that TA institutions greatly depended on external factors like the support of (changing) parliaments and/or ministries, volatile technology controversies, and shifting evaluation regimes in academia.

Junior TA practitioners were faced with the double burden of building up and nurturing academic identities as well as advisory roles and identities. Third-party-funded projects not only provided for their salaries, but also offered opportunities to develop TA specific expertise, skills, and culture. Because there was no standard TA project, participating in as many projects as possible was the logical way to achieve this. Yet, some (post-normal) skills seemed even impossible to convey or to acquire: “You need a kind of political wisdom, insider knowledge, a strategic mind, or whatever”, plus: “talent to keep specific collaborations alive and a certain grasp of other people, of other modes of procedure, it is much more personal”.

Though the junior practitioners had not joined ITA with the explicit aim of completing a PhD while there, over time, all developed an interest in or felt the need for further academic qualifications beyond “merely” contributing to contracted TA projects. But pursuing advanced degrees proved challenging. Writing a dissertation was “difficult to plan” due to ITA's status as a non-teaching academic institution, TA’s post-normal characteristics, and the diversity of one’s scholarly engagements. Moreover, the junior practitioner lacked role models on how to accomplish a PhD at ITA since the previous generation had already joined as established post-docs. Overall, they were left feeling inadequate and the impression of operating “in thin air”. This situation was even worsened – at least in the short run^20^ – by a newly launched academy career scheme that eliminated the possibility of a post-graduate career only within the institute.^21^

In sum, one would expect relatively strong socialization into, commitment to, and identification with the field because of the third generation's very early start into TA. The missing formal introduction to, initiation into, and demarcation of the field counteracted such identifications in the short run, as did the somewhat haphazard, constant hopping between very diverse projects and project teams and the double burden of building up and nurturing academic reputations as well transdisciplinary identities. In the long run, however, this very same project hopping could also result in a higher commitment to (or co-construction of) TA in more general terms and the TA community as such, that is, beyond a defined research area, individual practices, or distinct TA teams, probably also beyond ITA.

## Concluding Remarks: Generational and Disciplinary Specificities of Post-Normal Identity Trouble

In our study, we encountered a diversity of identities at ITA: from enthusiastic or pragmatic identifications with TA, to alternative identifications such as interdisciplinarians, disciplinary identities, and scholars in search of identity. Our study thus contributes to recent debates on the multiple, intersectional, fragmented, fluid, choreographed, strategic, and troubled identities in inter- and transdisciplinary settings. It adds to these debates along three dimensions: firstly, by providing insights on a transdiscipline that builds on substantive interdisciplinary integration; secondly, by focusing on an institute with a more than 30-year history, providing practitioners with the time horizon “required to learn an interdisciplinary culture and to grow into a research community” (Müller and Kaltenbrunner 2019) and even making generational differentiation analytically tangible; and, thirdly, by analysing an academic institution with a strong advisory focus, resulting in an ongoing recalibration of relations to science, the public sphere, and politics.

As to the first dimension, interdisciplinary integration, our findings support Marcovich and Shinn’s (2011) diagnosis of a “new disciplinarity” and provide further details for the case at hand. Disciplinary identifiers keep playing a significant role in scholarly identities at ITA, even if the overall identity regime has changed fundamentally and comes with its very distinct specificities in TA, further moulding the roles, functions, and ramifications of disciplinary identities and adding further identity options. For some ITA scholars, the original discipline remained a central identifier, not only in presenting themselves, but also in terms of networking and “careering”; but even the inter/transdisciplinarians occasionally felt strongly about their socialization, enculturation, and belonging to their initial disciplines. The TA practice mobilized manifold disciplinary expertise with diverse functions: Natural science and engineering expertise helped with better understanding the detailed workings of the assessed technologies and with reaching out to technoscientific actors; whereas social sciences and humanities expertise provided analytical methodologies and explanatory theories and allowed outreach to scholarly communities. Thus, former disciplinary identities played different roles in the interviewees’ accounts, with some specific characteristics for each generation.

The second dimension comprises the imminent time-horizon when studying a long-established institute with researchers of different ages, academic career stages, qualifications, and time spent at the institute. Other than the Cluster of Excellence researched by Bock von Wülfingen (2021) or interdisciplinary identities shaped by single interdisciplinary projects within a funding program (Felt et al. 2013, 2016; Schickowitz 2021), the focus on TA at ITA with its over 30 years history and its embedding in a transnational community allows for researching long-term trajectories of identity and belonging, including generational patterns and inter-generational relations. In these respects, our study points towards identity configurations that might not be found with the newest wave of short-term, singular institutionalizations such as clusters, collaboratories, or research hotels (for the latter, see Noël 2021). In other respects, we found similarities among all these cases, especially concerning how uncertain career futures have an impact on identities. Wenger’s (2000) notion that “identity has a trajectory” weighed heavily not only on identity options arising from TA practitioners’ past education, career, and belonging, it also concerned perceived options for “futuring one’s identity” and impinged on practitioners’ identification patterns in the present. Such options depended on assumptions about the institute’s future as well as the individual practitioner’s future career prospects at ITA. The recurring institutional crises, temporal contracts, “project hopping”, and limited or adverse career paths especially affected junior practitioners and protracted their scholarly identification. Thus, our study also emphasizes the relevance of temporal issues for scholarly identities. Building on Felt’s (2017) concept of chronopolitics that addresses “the politics of time governing academic knowledge production, work and evaluation”, we therefore claim that an important aspect of such chronopolitics lies in its grip on identity options.

With the long time-horizon of our empirical case, we can also confirm the finding of Bartlett et al. (2016) “that the cultural differences at play in interdisciplinary work are not only those of different disciplinary cultures, but also ‘generational’ differences” (ibid: 192). Given the interrelations we found between generational patterns and the institute's history, we suggest further developing the conception of generations put forward by these authors, drawing on Mannheim (1928). The manifestation of generations with distinct identities is not only related to generic developmental cycles like field emergence, institutionalization, and normalization/maturation. Instead, it is co-produced by internal as well as external factors, resulting in distinct “social locations” (“Generationenlagerungen”) and “generational units” (“Generationseinheiten”; for both, see Mannheim 1928). The three generations of ITA practitioners markedly relate to distinct socio-historical settings (the respective technology discourses of the time and place, the changing international TA landscape, the reforms of the Austrian Academy of Sciences, changing evaluation regimes, etc.) and split into generational subunits with respect to their disciplinary backgrounds. They experienced and coped in different ways with the generation-specific settings.

The third dimension comprises TA’s dual orientation towards academic output and societal impact, resulting in careful recalibrations of ITA’s relations to academia, the public sphere, and politics. Positioning oneself and ITA in this triangle (cf. Hennen and Ladikas 2009: 46) was recounted as a recurring issue in internal discussions at ITA and an integral part of professional TA identity, as were ongoing struggles over roles when bridging the science-policy boundary (Bauer and Kastenhofer 2019). Roles, norms, ethos, and societal missions and visions thus were decisive aspects of TA practitioners’ identities, or, more broadly speaking, their individual scholarly personae (see also Paul 2014). In these respects, our findings are reminiscent of Müller and Kaltenbrunner (2019). Our analysis of generational specificities allows us to better understand how each generation was affected by and coped with this situation. First-generation TA practitioners at ITA held permanent positions at the time of the interviews and did not feel pressured to focus solely on academic reputation;^22^ they tended to commit more strongly to advisory functions and activities and to identify more emphatically with TA. The academic orientation of a few “disciplinarians” from both the technosciences and the social sciences, however, coincided with uneasiness about too “activist” stances. The uncertain employment and affiliation of the second and third generations resulted in a need to further succeed academically and, thus, a more active academic commitment^23^, while also constantly recalibrating within the triangle of academia, the public sphere, and politics. At the same time, junior practitioners had less control over which projects they contributed to in which way, further amplifying this youngest generation’s identity troubles (cf. the findings of Felt 2013, 2016).

Our study emphasizes that the multiple roles, functions, and identities put considerable strain on community building and community stabilization in TA. At times, this resulted in the impression that there was more than one TA, that one could distinguish “TA in a stricter sense” from “TA in a broader sense”, or that there was no coherent TA identity at all. Such assessments were based on the implicit assumption that the identity of TA equalled the least common denominator of its practitioners’ identities and was at its strongest when all identified with TA in the same way. In contrast, we suggest an alternative understanding of TA‘s identity, as emerging from a complex ecosystem of individual identities, roles, and activities and thus even more than the sum of its practitioners’ individual identities. TA’s internal diversity allows for short-term reactions, adaptations, and adjustments on the individual as well as the collective level – a capability that is undoubtedly helpful for a boundary organization facing diverse environments and continuously changing external demands. A sense of belonging, institutional commitment, and collective identity (without being identical in all respects) can still be achieved by intense internal collaboration and exchange as well as a shared post-normal vision and ethos. The ITA has recently tried to strengthen such a shared vision by researching ITA’s advisory practices (Bauer and Kastenhofer 2019) or by discussing the normative dimension of TA (Nierling and Torgersen 2020). With the tremendous crises our society currently faces and the resulting necessity to keep up with the societal needs of the time, we deem it even more important that TA scholars self-consciously make use of their entire role and identity repertoire. It will lie in the hands of the current generation of TA practitioners to do so in a scientifically sound as well as societally robust way. This new mission might well form the core of future TA scholars’ identities.

## Footnotes

1 The line between inter- and transdisciplinarity is drawn in various ways in the literature. For the case at hand, we reserve the term “transdisciplinary” for a dual orientation towards knowledge generation via research practices and direct societal intervention via participatory and/or advisory activities; that is, for research that includes direct interaction with publics and/or decisionmakers. This categorization is to be understood as a text-specific, pragmatic solution rather than a generic theoretical proposition. If all variants are addressed, we use the combination “inter/trans/disciplinary”.

2 ITA self-presentation at https://www.oeaw.ac.at/en/ita/the-ita/about-us, last accessed 8 March 2022.

3 When speaking of contractors/clients in this text, we mean third parties that fund TA projects.

4 The second author has since left the institute for a university tenure track position.

5 In this respect, we could fortunately build on project-related data collected within PolITA and on a history of the institute prepared by Nentwich and Fuchs (2021) to celebrate the 175th anniversary of the Austrian Academy of Sciences.

6 All interview quotes have been translated from German into English by the authors. No interview numbers are provided so as to safeguard anonymity.

7 The latter quotes retrieved from the institute's homepage 25 January 2022.

8 One or two interviews within each generation relate to more than one type; with a few interviewees, the generational affiliation is also less clear than with the rest. With these caveats, the first generation comprises approximately six interviewees of which the half figure as “TA enthusiasts”, the second generation seven interviews of which more than the half figure as “strong interdisciplinarians” and the third generation seven interviews of which with more than the half figure as “flexible TA practitioners”.

9 Moreover, one can assume that those colleagues who had faced more severe identity troubles during the early phases of the institute had already left.

10 This label was used by two disciplinarians for staff members that had already left the institute before 2000. The assumption was that they had left because they regarded ITA and TA as “too toothless”.

11 The quest to place a higher priority on social relevance instead of scholarly excellence nevertheless also comes with its own challenges for social scientists (cf. Burawoy 2016; Jacob and Jabrane 2018; Abraham and Aulenbacher 2019).

12 We subsume natural scientists and engineers under the label “technoscientists” here so as to provide more anonymity.

13 The German term *Nestbeschmutzer* (literally: “nest defiler”) reflects a very pronounced expulsion from a community. It came up during one interview and related to the particularly challenging case of being viewed as someone who had not succeeded in technoscience and then moved on to denigrating this very field, as "someone who was taking revenge".

14 Science and technology studies, human ecology, sustainability science, or the like.

15 The reform of the academy was agreed on internally following severe pressure exercised by the Austrian Council for Research and Technology Development. This decision came only five years after the reform of Austrian universities by the Universities Act 2002. While the latter reform completed the universities development towards "full autonomy", the reform process of the academy challenged its autonomous leadership and internal leadership structure. Otherwise, both reforms shared a strong focus on an entrepreneurial approach and internationally competitive research.

16 The University Act 2002 also implemented a total limit of consecutive contracts of six years full time (eight years part-time). The academy mostly followed this rule, although the legal details remained unclear.

17 By then, interdisciplines such as science and technology studies, human ecology, or ecological economics were established as international fields with their own internationally renowned journals, associations, post graduate curricula, and dedicated university chairs.

18 All quotes from senior practitioners.

19 Performance Agreement 2012–2014 between the Federal Ministry of Science and Research and the Austrian Academy of Sciences, concluded on 4.11.2011, see oeaw.ac.at/fileadmin/NEWS/2011/pdf/Leistungsvereinbarung_2012-14.pdf (last accessed 28 February 2022), p. 9, translation by the authors.

20 Time spent at other institutions also furthered, in some cases, the self-esteem and identity formation of junior TA practitioners.

21 In this newly introduced scheme, junior scholars cannot directly move on to a senior position. If finishing their PhD when at the academy, they have to take a post-doc position at another research organization.

22 Like with the other two generations, their employment situations were very uncertain at the beginning of their TA career (albeit for slightly different reasons).

23 Only recently, TA practitioners have started mentioning that advisory activities can also harbour greater academic success, because they result in invitations to give keynotes at scholarly conferences or to collaborate in research projects. We take this as a hint that the academic regime is once again changing perceptibly, at least in some fields.
